# Findings from an expert focus group on psychotropic medication deprescribing practices for children and youth with complex needs

**DOI:** 10.3389/frcha.2024.1481446

**Published:** 2024-11-20

**Authors:** Laura Theall, Ajit Ninan, Melissa Currie

**Affiliations:** ^1^ Applied Research & Evaluation, Child and Parent Resource Institute, Ministry of Children, Community and Social Services, London, ON, Canada; ^2^ Division of Child and Adolescent Psychiatry, Western University, London, ON, Canada

**Keywords:** psychotropic medication, deprescribing, children and youth, complex needs, mental health

## Abstract

**Introduction:**

Psychotropic medication can be effective at stabilizing emotional and behavioural disturbances associated with physiological processes in children and youth. When medication benefits, indication or adverse effects are queried, deprescribing should be considered. Current guidelines for deprescribing are mainly for adults/elderly and largely theoretical, not practical, especially for polypharmacy.

**Methods:**

At a tertiary center for children and youth with complex emotional and behavioural needs, physicians on staff have expertise in conducting assessments of medication efficacy, side effect burden, and safety concerns. Deprescribing is routinely undertaken in the context of inpatient and outpatient services in partnership with children/youth and their families. A qualitative initiative leveraged the specialized deprescribing expertise of eight physicians (six psychiatrists and two pediatricians).

**Results:**

Emerging themes were medication review, timing, a stepwise approach, and setting conditions (inpatient and outpatient), with recurring subcategories of patient/family engagement as well as the underlying importance of continuity of care with psychosocial/behavioural supports.

**Discussion:**

The findings from this expert focus group serve as a step towards supporting prescribing clinicians in mindful deprescribing when medications are no longer in the best interest of young patients.

## Introduction

### Long-term psychotropic use and polypharmacy with children/youth

Psychotropic medications can be effective at stabilizing emotional and behavioural disturbances associated with physiological processes in children and youth. While use of medication for stabilization is appropriate practice in psychiatric treatment according to the American Academy of Child and Adolescent Psychiatry (AACAP; 1), children and youth with complex developmental, mental health and behavioural challenges are often prescribed psychotropic medications without a predetermined duration or plan for discontinuation resulting in potentially inappropriate long-term use (2). Consequently, psychopharmacology is used with children and youth with limited understanding of long-term effects. Possible adverse health consequences of young patients taking psychotropic medications over time can include excessive weight gain, reduced bone mineral density, growth impediments, thyroid issues, movement disorders, seizures, and metabolic syndrome increasing the risk for serious health conditions into adulthood (3–5).

Furthermore, prescription of numerous medications (i.e., polypharmacy) for children/youth with complex emotional and behavioural needs is common and can increase health risks. O'Brien and colleagues (6) recently shared the results of a 10-year analysis of psychotropic medication use for children/youth with severe behaviour challenges sampled across years who were receiving intensive behavioural therapy services. An average of 84% of the children/youth were taking at least one psychotropic medication, and polypharmacy rates remained high throughout the decade with a range of 58%–82% of the children/youth receiving services taking more than one psychotropic medication (6). Since each medication carries risk of side effects, polypharmacy can increase the likelihood of experiencing adverse reactions and drug interactions in the short-term, with health risks compounded with long-term use (3, 4, 7). A study by McLaren et al. (8) found that most caregivers understood why their child/youth was prescribed multiple psychotropics and more than half were aware of potential side effects, but only about a third were aware of potential long-term harm. Considering serious potential long-term adverse effects, Hoekstra & Dietrich (4) suggest prescribing alongside evidence-based behavioural interventions and limiting the use and duration of psychotropic medications with child and youth patients.

### Over-reliance on pharmacology, underuse of psychosocial interventions

Concern about excessive prescribing for children and youth has been growing. A review by Zito et al. (9) of United States (US) polypharmacy studies from the past 20 years shows an over-reliance on prescription of multiple psychotropic medications without psychosocial supports. Overprescribing is particularly high with vulnerable youth, such as in child welfare populations (10–13). In 2011, the US Government Accountability Office produced a report which prompted federal regulations calling for greater oversight and monitoring of prescribing practices for children/youth, specifically in foster care (14). To support this mandate, the Center for Health Care Strategies (CHCS; 15) shared a report in 2018 of improvement initiatives underway in several states to serve as examples for other states' monitoring and oversight efforts to recognize inappropriate polypharmacy, ensure informed consent of children/youth regarding prescribed psychotropic medications, improve monitoring of child/youth wellbeing and side effects, and promote consultations between social workers and child psychiatrists. The CHCS report indicated access to evidence-based psychosocial and behavioural interventions as a service system gap and suggested that increasing access to non-pharmacological supports should be a key objective for the future (15). Recognizing growing concerns about overprescribing, a recent review by Cosgrove and colleagues (16) urged psychiatrists to understand the strong influence that the pharmaceutical industry has had in this field leading to over-reliance on psychotropic medications.

### An illustration of overprescribing

Unfortunately, the medication route is easiest to access which puts prescribers in a difficult position. The current service system has excessive barriers to accessing non-pharmacological supports, which makes it difficult to achieve a reduction in prescribing practices (9, 16). Barnett and colleagues (2), through interviews with child and adolescent clinicians, found that with an overloaded mental health system, pressure from caregivers and schools, and barriers to continuity of care and psychosocial supports, it is unfortunately easy for children and youth to accumulate a complex medication regimen in a relatively brief amount of time. A fictional case example illustrates how overprescribing can occur:

V has been struggling with learning differences and internalizing symptoms for over 10 years. In kindergarten, she was considered bright and creative with immense potential. But in grade 1, she was labeled by her teacher as a “behaviour problem”. Sitting and focussing for the school day was impossible, handwriting was challenging, and learning math was difficult. In grade 2, V was diagnosed with ADHD by a private practice psychologist. An individualized education plan was developed by the school, and she was prescribed a stimulant by her family doctor. The drug suppressed her appetite and led to severe weight loss and extreme irritability over the following 6 months. Next, she was prescribed Risperidone, an atypical antipsychotic, to increase appetite and decrease irritability. Now adherent to 2 psychotropic medications and with academic accommodations in place, V was functioning better academically, but still experienced social challenges that tend to be comorbid with ADHD which were not addressed. She developed trouble sleeping, a potential side effect of both atypical antipsychotics and stimulants, for which she was prescribed Clonidine, a centrally acting alpha-agonist hypotensive agent. Her family moved to a new province when she was 14 years old, and V began experiencing bullying at school. She refused to attend classes, and developed symptoms of depression and anxiety for which she was prescribed Fluoxetine, a selective serotonin reuptake inhibitor (SSRI), by a new family physician. V began cutting and expressed suicidal thoughts and was admitted to an inpatient psychiatric facility.

With 4 psychotropic prescriptions at 15 years of age the psychiatrist-led multidisciplinary team began the task of reviewing appropriateness of the polypharmacy. The review clearly showed that medication discontinuation was indicated for several reasons: (1) medication(s) had been prescribed to treat potential side effects of other medications (e.g., Risperidone for stimulant-related appetite suppression and irritability, Clonidine for Risperidone-related and/or stimulant-related sleep disturbance); (2) an academic accommodation was put in place but no behavioural, family, or psychotherapeutic supports were implemented; (3) polypharmacy occurred without evidence-based indication; (4) severe decline in mental health condition occurred, potentially an SSRI-related adverse effect; (5) the patient had experienced disrupted continuity of care, lacking follow-up from prescriber(s). Now the team must decide which medication(s) to stop and create a plan to safely do so.

This fictional example illustrates how a snowball effect of polypharmacy can transpire for children and youth with neurodiversity and academic challenges when there are barriers to continuity of care, pharmacological management of side effects, and minimal non-pharmacological interventions and supports applied. The cumulative effect of overprescribing can go unchecked until the patient reaches a crisis point.

### Deprescribing within pediatric psychotropic regimens

When medication benefits, indication or adverse effects are queried, deprescribing should be considered. Bellonci (17), speaking on behalf of The American Academy of Pediatrics, defines deprescribing as “the systematic process of identifying and discontinuing medications in instances in which existing or potential harms outweigh existing or potential benefits.” The National Institute for Health and Care Excellence (18) provided guidance to consider deprescribing when there is not a benefit to the patient, dependence on the medication is becoming an issue, the condition is no longer a concern, the risk/benefit ratio is not in the patient's best interest, or there is a desire by the patient to discontinue the medicine. It is important to note that this recommendation is for adults and there is less guidance for children and youth with complex needs where communication challenges are common, especially when severe developmental disabilities are present.

Barnett and colleagues (2) gathered perspectives from primary care physicians, child and adolescent psychiatrists and psychiatric nurse practitioners on prescribing practices and experience deprescribing with children and youth. The results reflected a growing awareness of the detriments of polypharmacy, with individuals using personal standards for their own prescribing practices. Of concern was that less than half of the professionals had heard the term deprescribing. The researchers cast a wide net to obtain their sample of participating clinicians from across settings and regions within the US (2), therefore, it was not surprising that levels of experience and comfort with deprescribing differed within the sample. The study highlighted the need to prevent polypharmacy and to promote deprescribing as a best practice in pediatric care (2). While literature calling for reducing polypharmacy with children and youth is gaining momentum in recent years, deprescribing guidelines are still lacking (2, 3, 8–11, 13, 16, 19–21). Deprescribing frameworks have been designed and validated for geriatric populations such as in nursing home settings (22–24), but with limited generalizability to children and youth. Essential differences in deprescribing between elderly nursing home residents and children/youth with complex needs include consideration of long-term adverse effects of medication and life expectancy of patients, family partnership, and developmental changes that interact with efficacy of medication (5). Existing resources for deprescribing with child/youth patients are largely theoretical and untested, especially for polypharmacy (2, 9).

Off-label prescription with children and youth is common practice which complicates the task of establishing tools and guidelines for deprescription. For example, a review by Shahidullah et al. (25) cites prevalence of off-label psychotropic prescribing with children as high as 40%. In short, because most psychotropics are not intended for children under age 18, guidelines are lacking for this population. Therefore, deprescribing clinicians must seek guidance from a variety of disjointed sources when presented with a young patient carrying a burden from overprescribing, including reinterpreting prescribing guidelines to determine titration schedules to safely discontinue medications, especially those that have high risk of withdrawal effects.

### Purpose

To build awareness of pediatric deprescribing actions, the current initiative provides a qualitative analysis of an expert focus group for safely deprescribing psychotropic medication for children and youth using an approach based on constructivist grounded theory (26–28). At a tertiary care center for children and youth with severe emotional and behavioural challenges, physicians have extensive expertise in assessing internalizing and externalizing disorders and conducting reviews of medication efficacy, side effect burden, behavioural safety, and health concerns. This project was designed to leverage these physicians' unique expertise to demystify the deprescribing process and share knowledge grounded in practice.

## Method

### Participants and setting

A focus group of physicians took place at a large tertiary center that provides trauma-informed, highly specialized assessment, treatment, and targeted intervention services for children and youth from birth to age 18 with complex combinations of special needs, including developmental disabilities, autism, and severe behavioural, emotional and mental health challenges located in Ontario, Canada. The center was the first in Canada to adopt the Sanctuary Model® of organizational change, a trauma-informed care model which provides training and tools for staff and management to recognize the effects of adverse events in the past to build a healthier workplace that delivers better services (29). Trauma-informed principles underly every aspect of the culture and service delivery at the center.

Directly funded by the Ontario provincial government, the center serves an average of 314 inpatient and 1,816 outpatient children and youth per year, with an average of 2,058 unique individuals served per year (i.e., some patients receive both service types within a year). At this tertiary center, clinicians deliver care to referred children and youth with complex needs who require additional support above and beyond what is available in the community. Often patients are admitted to inpatient and outpatient services carrying multiple diagnoses with complicated psychotropic medication regimens that have accumulated from multiple prescribers. Diagnostic clarification and medication review are often priorities before other treatment recommendations are formulated. Adjusting medication regimens, which often includes deprescribing, is routinely undertaken for inpatient and outpatient children and youth in partnership with their families. All patients at the center receive services from comprehensive multidisciplinary teams which include physical, behavioural and psychosocial supports based on patient needs. The interprofessional patient- and family-centered clinical framework used at the center has been described by Ninan and colleagues (30). All thirteen physicians on staff were invited to participate in a focus group during a monthly medical staff meeting to provide guiding principles and thought processes they use in safely discontinuing medications with young patients.

### Procedure

A pre-survey was sent electronically to the physicians one month in advance of the planned focus group, with an 85% response rate (11/13 physicians). The pre-survey questions asked: 1. Are there a standard set of steps you follow when you deprescribe? 2. Is there anything about deprescribing you would like to learn more about? The responses helped facilitators to prepare for the engagement session. See Table 1 for responses.

**Table 1:** Results of a pre-survey administered to physician participants prior to the focus group.

Questions	Responses
Are there a standard set of steps you follow when you deprescribe?	• 36% No, the process is unique for each patient and/or medication • 27% Somewhat, but depends mostly on the medication type • 18% Somewhat, but depends mostly on the patient's needs • 9% Yes, I have a standard process I use for all patients and medication types • 9% Other: “both dependent on presentation and med type”
Is there anything about deprescribing you would like to learn more about? (free text responses)	• Indications for when to consider deprescription • More guidance about how to deprescribe, what colleagues are doing, and consensus guidelines to follow to minimize adverse effects (e.g., withdrawal effects such as akathisia) • Med/patient related issues and proper documentation • Outcome evidence (particularly with comorbidity such as primary Mood Disorders with other psychiatric diagnoses)

Physicians were informed that participating in the focus group was voluntary. The focus group took place virtually using Microsoft Teams with 8 physicians (2 pediatricians and 6 child and adolescent psychiatrists; 3 male, 5 female; 62% of physicians on staff). Reasons for non-participation were attributed to competing priorities and unavailability at the designated time. The focus group interview was exempt from human subject ethics review in accordance with the standards outlined by the Panel on Research Ethics Tri-Council Policy Statement (TCPS 2, Article 2.2b; 31). Specifically, this qualitative initiative did not include a research intervention and involved a group whose approach to medication management is standard practice at the center and not considered private.

A preliminary literature review was conducted to identify existing knowledge gaps and ambiguities on the topic of deprescribing with child/youth populations. The preliminary review in conjunction with the pre-survey informed guiding questions for the focus group. The focus group was conducted by an experienced facilitator (LT) employing a semi-structured interview format with the following prepared questions to guide discussion: What are reasons you deprescribe medications? How do you formulate a plan for deprescribing? What are the different considerations when deprescribing within inpatient vs. outpatient settings? What do you find most challenging about deprescribing? What resources do you currently turn to?

### Data analysis

The focus group was transcribed using Microsoft Teams' transcription feature, then verified for accuracy and manually cleaned by the facilitator (LT). Phrases were coded for themes through open coding followed by axial coding (32) using NVivo v.14 software by LT, then reviewed/approved by AN and MC.

## Results

**Figure 1:**
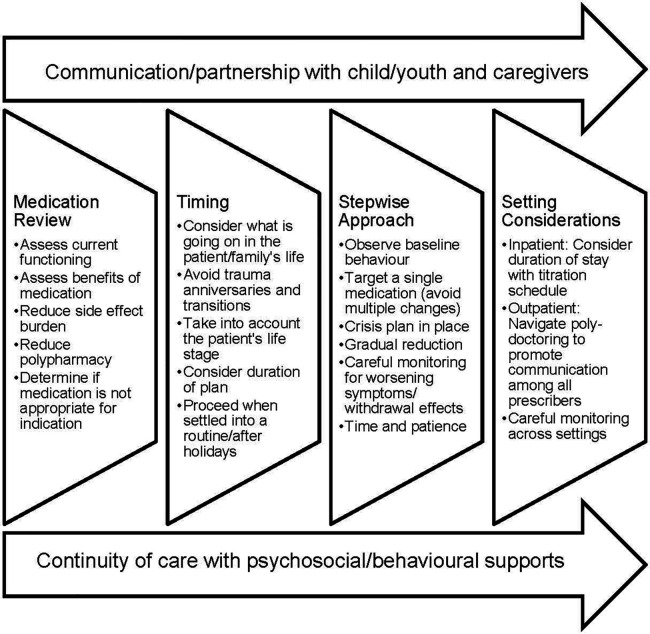
Expert-informed guidance to safely deprescribe psychotropic medications with child and youth patients.

The key themes central to safely deprescribing psychotropic medications with children and youth with complex needs that emerged from the focus group included medication review, timing, a stepwise approach, and setting conditions, with recurring subcategories of patient/family engagement as well as the underlying importance of continuity of care with psychosocial/behavioural supports to sustain deprescription and avoid re-prescribing. Figure 1 illustrates the findings from the focus group, as a step towards a psychotropic deprescribing framework for children and youth with complex needs.

### Medication review

The importance of reviewing patients' current medication regimen emerged as a prominent theme, with subcategories of patient/family engagement, reducing polypharmacy, assessing indication, presence of psychosocial supports, and assessment of adverse effects and benefits of medications. The physicians identified that simplifying medication regimens is a common goal to improve functioning. The group agreed with this statement by a psychiatrist describing their role in evaluating polypharmacy in new patients:

“Many of our kids come on a multitude of medications. And so our job is to decrease that side effect burden and to make sure that the medications they're receiving actually have indications and that the benefit of those medications outweigh the risks.”

During medication review, the physicians engage patients and caregivers for their perspectives on whether the current medications are working, or if there are concerns regarding efficacy or side effects. The request sometimes comes from caregivers or the patient themselves to discontinue medication. Polypharmacy in and of itself was discussed as a reason for deprescribing. For a child/youth to require so many medications to function was said to be quite rare and is usually an indication that other psychosocial or behavioural interventions are needed that have not been implemented.

In addition, the physicians noted that some conditions are minimally impacted with psychotropic medications, such as unresolved trauma and Fetal Alcohol Spectrum Disorder (FASD). The physicians routinely evaluate patients to determine if medications that have been prescribed are appropriate for diagnosed conditions. If it becomes evident that the prescription is for the wrong indication, for example to manage a situation or an environment medicinally, deprescribing is indicated and other non-medication behavioural or psychosocial modalities are considered.

### Timing

The theme of appropriate timing for deprescribing emerged mainly in relation to outpatient services, with subcategories of patient/family engagement, the life stage of the patient, and the duration needed for successful deprescribing. The consensus of the focus group was that applying a trauma-informed lens with mindful timing is key when formulating a plan for deprescribing. It is important to consider what is going on in the families' life (e.g., times of transitions, stress in the home). A pediatrician described their decision-making process regarding timing for deprescribing:

“One of the things that I take into account, and this is outpatient, is just what is going on in the families' life. So if they’re about to travel to Europe, do we put off any deprescribing or changes? I, you know this time of year, I try not to make too many changes. Christmas is coming up. Makes it very difficult to determine the outcome of a medication change, so I try to pick the middle of the fall or after Christmas. Once people are back into things, I try not to make changes in June because a lot of the kids don't do well in June, cause school is ending.”

Barring any difficult family circumstances, the group of physicians agreed that middle of fall is often optimal to implement deprescribing (i.e., October), after children/youth have settled into the school routine. The consensus was that following the late December school holidays is also a favorable time to implement changes.

The physicians shared lessons learned, sometimes the hard way, to avoid deprescribing around a trauma anniversary (e.g., a death of a loved one, major accident, episode of violence or abuse). Significant dates of adverse events, no matter how long ago, can trigger re-enactment causing emotions to be heightened with escalation of unsafe behaviours or amplify any adverse withdrawal effects during medication cessation. The physicians partner with caregivers in deprescribing planning to gain awareness of any adverse event anniversaries to avoid for inpatient and outpatient children and youth. Avoiding trauma anniversaries was discussed as particularly important for outpatient deprescribing, but awareness of significant adverse events can be helpful for inpatient deprescribing planning as well for trauma-informed patient-centered care. From a practical lens these variables must at least be considered as the deprescribing clinician and family assess the gradual response to a deprescribing plan when deciding if changes are medication-related or not. Avoiding deprescribing during such periods supports a less complicated background to draw conclusions.

Timing can also depend on the life stage of the patient. For example, deprescribing was said to be optimal in the second year of high school (e.g., grade 10) when major transitions are likely to be few rather than at entry (e.g., grade 9) or close to graduation (e.g., grade 12). The duration needed for successfully tapering off medications was discussed in relation to timing considerations with a trauma-informed lens and attention to upcoming life transitions on the horizon. For example, stimulants and alpha agonists can be safely discontinued in a shorter timeframe than SSRI medications. More discussion on duration occurred in relation to a stepwise approach below.

### Stepwise approach

A main theme of a stepwise approach involved in deprescribing emerged which included subcategories of patient/family engagement (including communication limits and managing expectations), baseline behaviour assessment, monitoring planning, crisis planning, and gradual reduction of medication dosage. While the path will look different for different types of medications and different children and youth, there are some common processes involved. The focus group conveyed the importance to first take inventory of what the child/youth's baseline behaviour is, to be able to assess later any changes related to withdrawing medication. For inpatient settings, this can be within the first two weeks of services to be able to assess behaviour changes during deprescribing, as stated by a psychiatrist:

“I don't do any medication changes for the first two weeks [of inpatient service] unless there's something glaring, right? Because I want to observe a baseline on those medications to be able to know what it changes.”

Allowing patients time to settle into the new setting with new routines also allows the physicians time to make baseline observations.

Ensuring caregiver and patient understanding and consent is an important part of the process. The physicians discussed how caregivers may take the child off medications without consulting the prescriber, which can cause serious withdrawal effects. Older youth may have opinions that differ from their caregivers and so the physicians spoke of navigating this to reach a consensus with the patient and family for how to move forward. Maintaining open communication with patients and caregivers about the improvements and adverse effects they may observe, as well as helping families to manage expectations and anticipate worsening symptoms or withdrawal effects with a crisis plan in place is essential to keep the deprescription process on track.

A stepwise deprescribing approach involves gradually decreasing a medication and avoiding multiple changes at once in order to carefully monitor and respond to worsening symptoms or withdrawal effects. The physicians raised awareness of cognitive and verbal limitations that may prevent children/youth from communicating discomfort from side effects and withdrawal effects, and therefore the deprescribing process may need to be slower for those vulnerable patients. The duration of action of the medication was also identified as a potential barrier. For example, antidepressants and antipsychotic medications take longer to discontinue safely to avoid withdrawal effects, and it takes time to observe how the patient is functioning during cessation. Generally, the more time that is used to assess a response to a dose change, the more confidence exists for drawing a conclusion based on a solid pattern of data. Daily dose reductions, even for short acting stimulants, may provide insufficient time to establish a solid pattern of observational data. The entire process takes time and patience for the child/youth, family, and all involved clinicians.

### Setting conditions

Because the physicians in the focus group deliver inpatient and outpatient services, the central theme of setting conditions emerged, with subcategories of, again, patient/family engagement, monitoring benefits and challenges, community supports, care fragmentation, and duration of stay limitations.

#### Inpatient

The physicians reported increased confidence deprescribing within the inpatient setting due to benefits of close observation and monitoring in a more consistent and controlled environment. For example, a psychiatrist described the deprescribing opportunity during inpatient admission as follows:

“Inpatient, I have the luxury of lots of observation, so I can feel more confident, they're with me. I have lots of data. Let's get this done while I have them.”

The physicians reported that they were able to rely on inpatient staff for important observations to inform their planning. Direct care staff at this inpatient center are trained to recognize and document potential adverse effects related to medications, as described a decade ago by Ninan et al. (33). Direct care staff are also trained to recognize withdrawal effects and escalating behaviours and to safely intervene to prevent the child/youth from entering a crisis state. Along with direct care staff and physicians, there is a multidisciplinary team model used at the center (e.g., psychology, social work, occupational therapy, speech and language therapy). With a multidisciplinary team, psychosocial interventions are implemented while medication changes take place. Even so, partnership with the family is extremely important for inpatient children/youth as it is common for patients to go home on the weekends or for holidays. It is essential for families to be on board to maintain the deprescription process and report back to clinicians on their observations in the home/community environment. The physicians cautioned that while the process can be going in the right direction on the unit, when the patient goes home for the weekend or an extended leave of absence over a holiday, potential environmental stressors and caregivers being unprepared to respond to escalating behaviours can derail deprescription plans. Ensuring caregivers are prepared and have strategies to manage the plan at home is important.

Limited duration of stay was said to present significant challenges. Because the general rule is to make a single medication change at a time, it can be frustrating for physicians to be limited in their capacity to reduce polypharmacy to a satisfactory level. For example, the average length of stay is generally up to 3 months at the center where the physicians practice, but some medications such as antidepressants and antipsychotics take longer than others to slowly taper off, and so the deprescription must continue in the community for certain long-acting psychotropics, such as antidepressants (e.g., fluvoxamine). This leads to the challenges of deprescribing on a time-limited inpatient basis.

#### Outpatient

While inpatient settings are more amenable to deprescribing, waitlists tend to be long and residential center locations may be far from home. Outpatient services are often more accessible than inpatient services. Physicians reported decreased confidence discontinuing medication on an outpatient basis in the community for several reasons: (1) there is less monitoring in uncontrolled home environments with unknown factors and situations; (2) it is more difficult to find community service providers to implement psychosocial and behavioural therapies for children and youth with complex needs, especially those with severe developmental challenges; and (3) there can be pressure from caregivers for medication to address safety concerns related to problematic behaviour. The pressure outpatient prescribers can feel which can lead to overprescribing and a reluctance to deprescribe was succinctly described by a psychiatrist:

“In the community, it's really hard to figure out what to do with these complex children when all you're going by is one visit every one to three months. And those visits are based off of probably one parent’s recollection of the last one to three months. And so it’s very easy for it to become, ‘Oh, he’s still struggling, let’s increase the dose. Let’s add one more thing. Let’s try this. Let’s try that.’ And then kids wind up on these very complicated cocktails of medications that may ultimately be causing side effects, may not be helpful, or could be making the problem worse. I always feel like my role is to try to be the child’s advocate for being on the least amount of medications possible to have the best response.”

The other physicians in the group agreed with this statement and reported often being in situations where the most helpful course of action is to deprescribe, which then requires an engagement process with families.

Although it can also be a challenge, a benefit of deprescribing on an outpatient basis is the opportunity to engage families and patients as true partners in the treatment process. The prescribers rely greatly on caregivers to be their informants. A common barrier can be that caregivers are unwilling to consider taking children/youth off medications because they are fearful of worsening symptoms. With patients at home, managing caregivers' fears and expectations is essential, along with collaborative planning. Considering the complexity of planning to deprescribe on an outpatient basis, a psychiatrist stated:

“So thinking about what is going to be and planning with the parents and the young person…what to expect? How to monitor and who to connect with? You know, what might be the next step? Condition A gets worse versus condition B, C, and D right? Because we are talking about comorbidity and we're talking about polypharmacy, that’s what [we] usually do from the outpatient perspective.”

The physicians reported working with caregivers to understand how to monitor the child/youth's condition and who to connect with if behaviour escalates or adverse effects occur (e.g., when to go to the local emergency department). Also, planning what might be the next steps if one condition improves while another worsens is important considering comorbidity of indications and polypharmacy are often involved. Working with caregivers at the start can facilitate more accurate assessment of the deprescribing plan and help prescribing clinicians to make informed decisions in the patients' care.

Care fragmentation resulting from “poly-doctoring” can present significant challenges, as described by a pediatrician during the focus group:

“We've … touched on this a little bit, the polypharmacy, but also the … concept of poly-doctoring. So there’s a big difference between inpatient and outpatient care and that being the number of doctors that may be involved and maybe prescribers on the outpatient end is much more of a consideration than the inpatient when you have that child. I find that’s a big issue with outpatient clients is to keep track of all the potential prescribers.”

In the community, a patient might be under the care of several prescribing clinicians and so bridging communication with these professionals is essential to a successful deprescribing plan. Coordination of care was said to be a major difference between inpatient and outpatient settings with the number of doctors that may be involved with each patient. The physicians suggested to speak directly with other involved prescribing clinicians to communicate the deprescribing plan orally and follow up with written communication.

## Discussion

There's a growing awareness that deprescribing is an essential component of best practice in prescribing psychotropic medications, but guidance in the literature is sparse (19, 34, 35). Because of the complex patients seen at this tertiary center and the wealth of experience reducing polypharmacy, the physicians engaged in this focus group are uniquely positioned to contribute to this knowledge base. Findings from the focus group contribute to a foundation on which to begin to build safe deprescribing practices for children and youth. As illustrated by Figure 1, best-practice themes emerged that include medication review for efficacy and side effect burden of the current regimen, attention to appropriate timing, a planful stepwise approach, and consideration of the setting in which the child/youth is receiving care. Overarching is the need for effective communication with the child/youth patient and caregivers. Underlying sustained deprescribing is ensuring continuity of care and access to psychosocial and behavioural supports.

Medication review emerged as a theme early in the focus group discussion as central for best-practice care for children and youth with complex needs, and as the premier step in deprescribing. Conducting a thorough medication review involves taking inventory of the current and past medication regimen, appraising the current regimen in regard to the child/youth's history and diagnoses, investigating adherence to the regimen, assessing the child/youth's current functioning in relation to the benefits of medication(s) and experienced side effects, and evaluating the number and combinations of prescribed medications for appropriateness (i.e., assess polypharmacy and contraindications) (1, 36–38). Benefits of conducting a thorough medication review include improved symptom management, ensuring medication safety, and optimizing treatment that accounts for developmental changes (36–38). The focus group of physicians expressed that taking the opportunity to do a thorough medication review is key in patient-centered care planning to improve child/youth functioning. The results of the medication review are essential to inform deprescribing planning that includes careful timing and a stepwise approach.

A recurring theme subcategory was patient/family engagement. Overprescribing often results from the pressure that prescribers feel from families for any available help (2). Ensuring that caregivers feel supported during the deprescribing process is essential. It may be appropriate to draw generalities from the literature focused on deprescribing with adults with intellectual disabilities in this regard. In a review, Adams and colleagues (39) found positive health benefits to deprescribing psychotropic medications without increasing problem behaviours during the process when slow tapering plans were in place. But evidence of re-prescribing at follow-up timepoints was found to address resurgence of challenging behaviours. Similarly, in children/youth with developmental and communication challenges, there is the potential for problematic behaviours associated with agitation due to discomfort from withdrawal effects that can mimic a relapse of unsafe behaviours from the psychiatric condition, which makes a case for psychosocial support during deprescription (39). Planning for this possibility can avoid the assumption that the medication is needed and prevent re-prescribing (39, 40). Adams et al. (39) also reported relevant studies were lacking in describing how families and the patients themselves were engaged in deprescribing plans and processes and noted that engagement in shared decision making is essential for patient-centered care. A review by Chock et al. (41) indicates a willingness among (adult) patients and caregivers to proceed with deprescribing plans and states that their collaboration is important for patient safety.

Sourcing other mental health non-pharmacological treatments is essential to successful discontinuation of psychotropic medications (42). Zito and colleagues (9) cite a flawed endorsement of a biological model of mental health care that disregards needed psychosocial interventions as at the root of the problem of overprescribing. Levin (43) described successes in tapering off psychotropic medications with extremely aggressive children and youth by recognizing the patients' symptoms as related to early experiences of trauma, employing psychodynamic approaches to treatment, and helping patients to develop coping skills. Levin attributed successful deprescribing to working with patients at their own pace by engaging them to determine openness to therapy and readiness to discontinue medications (43). Cosgrove et al. (16) call for psychiatrists to bring contextual causes of mental health crises (e.g., developmental trauma) to the forefront of practice, adopting a “gentle medicine” approach when treating psychological distress. Practicing gentle medicine means endeavouring to help patients make changes to improve their lives and situations and prescribing less, originally described by Stegenga (44). In place of relying heavily on pharmaceuticals to treat individuals with mental health challenges, addressing systemic inequities that contribute to mental health problems (e.g., poverty, racialized communities, housing challenges, food insecurity) could prove to be more effective on a wider scale and longer term for mentally healthy individuals and communities (16). Cosgrove et al. boldly state, “Thus, if psychiatry is to take the idea of gentle medicine seriously, the field would need to acknowledge the lack of effectiveness of many psychotropics, acknowledge their harms, and embrace a tolerance for uncertainty, while improving the lives of some people, these agents are simply not the magic bullets we hoped they would be” (16, p04). A commitment to rely less on medications to improve child/youth functioning was evident in the focus group discussion with physicians.

### Limitations

A limitation of findings is the small sample size of eight participating physicians from a single tertiary care center. Also, while the complexity of patients at the center may be a benefit, deprescribing considerations and practices may differ with less complex patients in community clinic settings. An additional limitation is that quantitative data on deprescription rates and titration schedules at the center to support the physicians' discussion points was not available. At the time of this writing, the pharmaceutical data infrastructure at the center did not allow for parsing types of medication orders without a manual review process.

### Future directions

This initiative represents a step towards future development of guiding principles for deprescribing in children and youth with complex needs. Future research in this area should be directed towards developing a pediatric deprescribing framework and producing medication-specific and patient/family-centered guidelines to promote safe deprescribing practices with vulnerable children and youth with complex needs. A comprehensive initiative of this magnitude will require collaboration between academic researchers and child/youth treatment centers with well-funded rigorous research on actual deprescribing practices. While randomized controlled trials are considered robust, clinical decisions in practice are made in the best interest of the patient, without withholding care for the sake of research (45). With improvements in electronic health records systems comes an opportunity to leverage existing data to determine commonalities and patterns in titration schedules, evaluate outcomes of deprescribing, and gather recommendations for non-medication supports to safely simplify or discontinue medication when the risk/benefit ratio is no longer in a child or youth's best interest.

Future investigations are also needed to document the rate and practice of deprescribing with specific populations where polypharmacy is a high concern, such as in foster care and group homes (10–13, 19). Additional focus on the phenomenon of poly-doctoring would be beneficial, to explore methods to improve communication between prescribers for better outcomes for children and youth. The current setting has the benefit of physicians on staff. In the community, continuity of care can be a barrier to ensuring children/youth receive the least medication needed. The child/youth sector would benefit from more attention to bridging care gaps and improving service provider communication.

Addressing current overprescribing practices is emerging as a priority in the field, but attention should also be given to training future clinical prescribers in determining when deprescribing is indicated. A study by Poots et al. (37) found that while medical profession trainees were gaining familiarity with the concepts of medication review and polypharmacy, knowledge of deprescribing appeared to be a curriculum gap. Training new clinicians in mindful prescribing should include integrating deprescribing planning into treatment plans at the prescription stage. For example, AACAP (46) provides prescribing guidelines for obsessive compulsive disorder (OCD) that include prescribing SSRI medication in a slow stepwise fashion, reaching optimal dose with a length of time for the patient to continue at the optimal dose, and subsequent deprescribing. Including deprescribing planning at the prescription stage could potentially improve continuity of care and prevent adverse health effects by avoiding long-term psychotropic use.

As deprescribing gains wider practice, future research should investigate disparities using socio-economic and identity-based data. For example, an increased rate of prescribing certain psychotropic medications with higher risk of serious adverse effects to children and youth from lower income families has recently been documented (i.e., benzodiazepines and antipsychotics instead of SSRI's; 47). Just as current research is bringing *prescribing* disparities to light, future research should investigate practices of *deprescribing* with an equity-based lens as well.

## Conclusion

While psychotropic medication has been an effective component in the treatment of severe emotional and behavioural disorders for many decades, the concept of deprescribing medications, or simplifying a child/youth's medication regimen, is gaining momentum as an important component of best practice for evidence-based care. Deprescribing is more than a buzzword, it is an essential component for consideration in treatment planning to support children and youth with complex needs to reach their full potential.

## Data Availability

The datasets presented in this article are not readily available. A transcript of the engagement session may be available upon request if approved to share by the Director of the center. Requests to access the datasets should be directed to Laura Theall, laura.theall-honey@ontario.ca.
